# Perioperative temporary microaxial left ventricular assist device support in patients with low ejection fraction undergoing coronary artery bypass grafting

**DOI:** 10.3389/fcvm.2026.1911213

**Published:** 2026-09-28

**Authors:** Allyson Howard-Green, Deevya Raman, Mark Cordell, Spencer Tingey, Jake DuPlooy, Akalya Villenthi, Timothy Craven, Neal Kon, Adrian Lata, Karuna Puttur Rajkumar, Olivia Gilbert, Bartlomiej Imielski

**Affiliations:** 1 Wake Forest Baptist Medical Center, Department of Cardiothoracic Surgery, Winston-Salem, NC, United States; 2 Wake Forest Baptist Medical Center, Department of Academic Biostatistics and Data Science, Winston-Salem, NC, United States; 3 Wake Forest Baptist Medical Center, Department of Cardiothoracic Anesthesia, Winston-Salem, NC, United States; 4 Wake Forest Baptist Medical Center, Department of Cardiovascular Medicine, Winston-Salem, NC, United States

**Keywords:** CABG, coronary revascularization, heart failure, impella 5.5, temporary LVAD

## Abstract

**Purpose:**

Despite advances in mechanical circulatory support (MCS), outcomes in high-risk coronary artery bypass grafting (CABG) with low ejection fraction (EF) remain unclear. We evaluate short-term outcomes associated with temporary microaxial flow pump (TMFP) support in high-risk patients undergoing CABG during the same hospitalization.

**Methods:**

This retrospective study reviewed 111 patients who underwent CABG with Impella 5.5 or LD TMFP support during the same hospitalization between April 2018 and January 2025. Patients receiving extracorporeal membrane oxygenation (ECMO) were excluded. Baseline demographics, procedural characteristics, and postoperative outcomes were collected. Statistical analyses were performed using R version 4.4.0 with significance defined as *p* < 0.05.

**Results:**

The cohort (*n* = 111) had a mean age of 69 years and was 75% male (83/111). Comorbidities documented prior to index hospitalization included hypertension (85%, 94/111), diabetes mellitus (57%, 63/111), coronary artery disease (CAD) (47%, 52/111), and at least one prior cardiac surgery or intervention (31%, 34/111). Support was planned in 30% (33/111) of cases and unplanned in 70% (78/111). 30-day survival was 89.2% (99/111) and 1-year survival of 74.8% (83/111). No statistically significant differences in mortality were observed according to performance of concomitant procedures or operative time periods (2018–2020 vs. 2021–2025).

**Conclusion:**

In high-risk low-EF patients undergoing CABG, TMFP support during the same hospitalization was associated with acceptable 30-day and 1-year survival outcomes among patients considered unsuitable for PCI and/or advanced therapies at the time of surgery. Mortality was not significantly different among patients undergoing concomitant procedures or between planned vs. unplanned TMFP placement. These findings support TMFP's growing role in complex coronary surgeries and warrant further studies to define optimal application and surgical candidacy.

## Introduction

Coronary artery bypass grafting (CABG) remains the gold standard for revascularization in patients with advanced CAD and impaired left ventricular (LV) function. However, patients with severely reduced ejection fraction (EF) continue to experience substantial perioperative risk, including hemodynamic instability, perioperative myocardial injury, renal dysfunction, and increased mortality (1).

Despite these risks, growing evidence suggests that CABG improves survival and quality of life when compared to percutaneous coronary intervention (PCI) in appropriately selected low-EF patients (2). Nevertheless, perioperative mortality and morbidity remain elevated, underscoring the need for adjunct strategies to mitigate risk in this vulnerable population (3). MCS devices are increasingly applied as assistive devices in the setting of high-risk cardiac surgery. Particularly, the Impella (Abiomed, Danvers, MA) family of devices have gained popularity because they can directly unload the left ventricle, increase cardiac output, reduce myocardial stress, and enhance organ perfusion in both perioperative and cardiogenic shock settings (4–6).

In the setting of low-EF CABG, the Impella offers a new adjunct to traditional pharmacologic and intra-aortic balloon pump support by stabilizing hemodynamics, allowing for complete revascularization, and reducing myocardial workload during the perioperative period. Despite the growing role of Impella in complex cardiac surgeries, data remains limited regarding optimal timing of device placement (preoperative, intraoperative, or postoperative) and outcomes across larger patient series with longer follow-up (5, 7).

To address these gaps, we conducted a retrospective single center study of patients who underwent low EF CABG with Impella support during the same hospitalization. We evaluated short-term survival, length of stay, and myocardial recovery, with the goal of clarifying the role of Impella support in expanding surgical candidacy among high-risk, depressed EF patients and informing perioperative management decisions.

### Material and methods

#### Study design

This was a single center, retrospective observational study conducted at a tertiary care center with an advanced heart failure program. All adult patients undergoing CABG with Impella support during the same hospitalization were included. The primary outcomes were 30-day and 1-year mortality. Secondary outcomes included length of stay and myocardial recovery. Patients requiring ECMO support were excluded.

#### Study population and sample size

The study included 111 patients who underwent CABG and received Impella 5.5 or LD between April 2018 to January 2025. Patients were eligible if they received Impella placement preoperatively, intraoperatively, or postoperatively during the same admission as their CABG operation. This study was designed to evaluate perioperative Impella support among high-risk CABG patients requiring temporary MCS during the index hospitalization, regardless of timing of device initiation. Patients with ischemic cardiomyopathy and reduced LVEF (left ventricular ejection fraction) underwent multidisciplinary heart team evaluation to determine the most appropriate treatment strategy, including medical therapy, PCI, CABG, or advanced heart failure therapies with simultaneous assessment of surgical candidacy based on predicted STS risk score, surgeon judgement, overall functional status, and rehabilitation potential. Cases were classified as “planned” when Impella implantation was part of the preoperative plan before the patient entered the operating room and as “unplanned” when the decision to place an Impella was made intraoperatively or postoperatively based on patient's clinical status.

#### Surgical procedures

Standard cardiac surgical protocols were followed, with all cases performed using on-pump arrested cardiopulmonary bypass. A direct aortic Impella insertion approach was preferred (106/111, 95.5%) for device placement at the time of CABG with the rest performed through a right axillary approach (5/111, 4.5%). The direct aortic approach consisted of a 10 mm Dacron graft anastomosed in an end-to-side fashion to the ascending aorta, tunneled supra-clavicularly, with either transesophageal and or fluoroscopic guidance for pump placement. Pre- and post-operative insertion was performed via axillary artery cut down, anastomosis of an 8 or 10 mm graft, and fluoroscopically-guided placement.

The indication and timing of pre, intra, and post-operative Impella placement were determined by multidisciplinary clinical assessment and varied based on timing of cardiogenic shock and clinical presentation. For patients who received pre-operative or post-operative Impella support, the decision was made based on decompensated invasive hemodynamics suggestive of low output heart failure despite inotropic support. Intraoperative placement of MCS, including Impella support, was considered in patients who failed to wean from cardiopulmonary bypass due to persistent ventricular dysfunction and inadequate cardiac indices despite inotropic support with or without concomitant vasoplegia.

Anticoagulation management during Impella support was guided by institutional protocols and duration of device support. Short-term Impella support, defined as <72 h, was typically managed with bicarbonate-based purge solution without systemic anticoagulation. Patients requiring prolonged Impella support, such as those undergoing evaluation for advanced therapies, received systemic bivalirudin anticoagulation while continuing bicarbonate-based purge therapy.

#### Data collection

Clinical data were collected retrospectively from the electronic medical record (EMR), including demographic information, echocardiographic/catheterization findings, laboratory values, and procedural details. Postoperative acute kidney injury (AKI) was defined as an increase in serum creatinine by ≥1.5 times baseline within 7 days. Cardiogenic shock was categorized according to the Society of Cardiovascular Angiography and Intervention (SCAI) Shock Classification with Advanced Cardiogenic Shock defined by SCAI Stage D or E (8).

Comorbidities were manually abstracted from documented preexisting diagnoses in the medical record at the time of admission. Patients presenting with newly diagnosed CAD during the index hospitalization were not classified as having a prior history of CAD unless such a diagnosis had been previously made. As a result, reported comorbidity rates reflect known preadmission medical history and may underestimate the true prevalence of disease in patients presenting emergently or with limited prior medical follow-up. Prior myocardial infarction (MI) was categorized based on the timing relative to CABG as within 6 weeks or greater than 6 weeks before surgery. Patients with both recent and remote MI were classified based on the timing of the most recent event.

The Society of Thoracic Surgeons (STS) Predicted Risk of Mortality (PROM) was obtained from the procedure-specific STS risk calculator for each patient's index operation. For patients undergoing concomitant valve or aortic procedures, these procedures were incorporated into the STS risk calculation, and the reported mean STS PROM represents the average of the individual patient-specific estimates across the cohort.

For patients transferred from an outside hospital, admission was defined as arrival at our institution. Admission variables represent the earliest available data obtained after admission to our institution. Transferred patients were not analyzed separately and were considered part of a single continuous hospitalization.

Bleeding-related outcomes were defined as a hemoglobin decline ≥5 g/dL accompanied by red blood cell (RBC) transfusion occurring more than 24 h after any surgical procedures. Patients who died within 24 h of their final surgical procedure were excluded from this bleeding analysis, but remained for other outcomes, including 30-day and 1-year mortality (9).

#### Statistical analysis

All statistical analyses were conducted using R (version 4.4.0). Patient characteristics including demographics, preoperative and baseline variables, CABG procedure details, duration of Impella support, hemodialysis characteristics, and clinical outcomes (length of hospital stay; 1-month and 1-year mortality; 6-month and 1-year rehospitalization) were summarized using frequencies and percentages for categorical variables and medians with interquartile ranges (IQR) for continuous variables.

Mortality outcomes were assessed in relation to several factors: early experience (2018–2020) versus later period (2021–2025), presence of concomitant procedures, planned versus unplanned Impella placement, and presence of advanced cardiogenic shock at admission. Statistical comparisons of categorical outcomes were conducted using Pearson's Chi-squared test or Fisher's exact test as appropriate. Continuous outcomes were assessed using the Wilcoxon rank-sum test. Preoperative EF was compared with discharge EF using the Wilcoxon signed-rank test for paired data.

## Results

### Baseline demographics and admission characteristics

Baseline demographics and admission characteristics are summarized in Table 1. The median age of the cohort was 69 years (IQR, 59–74), and 83 patients (74.8%) were male. Prior MI was present in 59 patients (53.2%); 26 patients (23.4%) had MI within 6 weeks of CABG, while 33 patients (29.7%) had MI greater than 6 weeks before CABG. Mean STS PROM for the cohort was 15.7%.

**Table 1:** Baseline demographics and comorbidities and preoperative characteristics.

Characteristics	*N*	N (%) or [IQR]
Age (years)	111	69 [59, 74]
Gender	111	
Female		28 (25.2)
Male		83 (74.8)
BMI	111	27.8 [24.6, 32.0]
Hypertension	111	94 (84.7)
Atrial Fibrillation	111	8 (7.2)
Coronary Artery Disease	111	52 (46.8)
Myocardial Infarction		
No Prior MI	111	52 (46.8)
Prior MI (onset < 6 weeks)	111	26 (23.4)
Prior MI (onset > 6 weeks)	111	33 (29.7)
STS PROM	111	15.7%
Peripheral Vascular Disease	111	20 (18.0)
Chronic Renal Insufficiency Stage III (eGFR 30-59) or IV (eGFR < 30)	111	26 (23.4)
Was the patient dialysis dependent prior to presentation?	111	7 (6.3)
Diabetes	111	63 (56.8)
COPD/Emphysema	111	31 (27.9)
Obstructive Sleep Apnea (OSA)	111	22 (19.8)
Cerebrovascular Disease (CVA, TIA)	111	18 (16.2)
At Least One Prior Cardiac Surgery/Procedures	111	34 (30.6)
Prior PCI	111	28 (25.2)
Prior CABG	111	3 (2.7)
Prior Aortic Valve Replacement	111	3 (2.7)
Prior Pacemaker Placement	111	7 (6.3)
AST	98	25 [18, 53]
ALT	99	23 [15, 32]
Creatinine Level at Admission	108	1.19 [0.90, 1.58]
BUN	107	23 [17, 33]
Inotropes/vasopressors at admission	111	18 (16.2)

Laboratory values reflect most recent measurements obtained prior to Impella placement. Values obtained more than 30 days before Impella placement were excluded. Values/criteria for at-admission criteria include data from the first 24 h of admission. Laboratory data were missing for up to 27 patients. Prior MI was categorized as occurring within 6 weeks or >6 weeks before CABG based on the timing of the most recent MI event. Comorbidities reflect documented preexisting diseases prior to admission and do not include any newly diagnosed conditions identified during the index hospitalization.

Comorbidities were common and included hypertension, diabetes mellitus, chronic kidney disease (CKD), COPD, and at least one prior cardiac surgery or intervention. Baseline laboratory values obtained prior to Impella implantation are detailed in Table 1.

### Markers of shock

Markers of shock are presented in Table 2. Patients without advanced cardiogenic shock (*N* = 97 SCAI Stage A 78.4%, *n* = 76; Stage B 12.4%, *n* = 12; Stage C 9.3%, *n* = 9) had a median time from admission to CABG of 5 days (IQR, 2–8), allowing for preoperative optimization. In those, inotrope or vasopressor use was infrequent (4.1% at admission/transfer and 6.2% preoperatively).

**Table 2:** Markers of shock at admission and immediately prior to CABG, stratified by presentation with advanced cardiogenic shock.

Characteristics	Presenting in Advanced Cardiogenic Shock (*N* = 14)	Not Presenting in Advanced Cardiogenic Shock (*N* = 97)
Admission BNP	1,707 [932, 1802]	1,099 [634, 1830]
Admission Lactate	4.9 [1.41, 6.2]	1.48 [1.0, 1.8]
Admission SCAI stage		
A	0 (0)	76 (78.4)
B	0 (0)	12 (12.4)
C	0 (0)	9 (9.3)
D	11 (78.6)	0 (0)
E	3 (21.4)	0 (0)
Admission Inotropes/vasopressors	13 (92.9)	4 (4.1)
Admission Vasoactive agents/MCS		
0 drugs/devices	0 (0)	88 (90.7)
1 drugs/device	0 (0)	9 (9.3)
2 drugs/devices	2 (14.3)	0 (0)
3 drugs/devices	6 (42.9)	0 (0)
≥ 4 drugs/devices	6 (42.9)	0 (0)
Time from Admission to CABG (days)	0 [0, 3]	5 [2, 7.75]
Pre-op BNP	1,239 [449, 1633]	735 [371, 1412]
Pre-op Lactate	1.16 [1.02, 1.66]	0.87 [0.7, 1.17]
Pre-op Inotropes/vasopressors	13 (92.9)	6 (6.2)

Advanced cardiogenic shock was defined as SCAI stage ≥ D at admission. Admission laboratory values reflect most recent measurements obtained following admission; values obtained prior to admission were excluded. Patients receiving inotropic and/or vasopressor support within 24 h of admission were classified as receiving support at admission. Preoperative laboratory values reflect most recent measurements obtained within 24 h prior to CABG; values obtained greater than 24 h before surgery were excluded. Patients receiving inotropic and/or vasopressor support within 24 h prior to surgery were classified as receiving preoperative support. Degree of vasoactive/MCS support was defined as the total number of vasoactive medications (vasopressors and inotropes) and MCS devices required within 24 h of admission; therapies initiated intraoperatively or postoperatively, when CABG occurred within the first 24 h of admission, were not included in admission support or SCAI staging classification. Laboratory data was missing for up to 76 patients at admission and 65 patients preoperatively. Percentages might not total 100% due to rounding. Values are presented as N (%) or median [IQR].

In contrast, patients presenting in advanced cardiogenic shock (*N* = 14, SCAI Stage D 78.6%, *n* = 11; Stage E 21.4%, *n* = 3) underwent more urgent surgery with a median time from admission to CABG of 0 days (IQR 0–3). In the latter group, most patients required vasoactive support at admission (92.9%), and all required at least two vasoactive agents and/or MCS devices with 85.8% requiring three or more therapies. Most patients with advanced cardiogenic shock continued to require inotropic or vasopressor support preoperatively (92.9%).

### Operative details

Operative characteristics are presented in Table 3. Grafting complexity varied, with the majority of patients receiving three or more grafts. Specifically, 3 patients (2.7%) received a single graft, 10 (9%) received two, 38 (34.2%) received three, 50 (45%) received four, and 10 patients (9%) received more than four grafts. Of the 111 patients, 86 patients (77.5%) underwent isolated CABG, while 19 patients (17.1%) underwent concomitant procedures, including CABG with aortic valve repair/replacement (AVR) (*n* = 8), CABG with mitral valve repair/replacement (*n* = 7), CABG with aortic aneurysm repair (*n* = 2), and CABG with AVR conduit (*n* = 2).

**Table 3:** Operative characteristics and echocardiographic parameters.

Characteristics	*N*	*N* (%) or [IQR]
Number of CABG Grafts	111	
1		3 (2.7)
2		10 (9.0)
3		38 (34.2)
4		50 (45.0)
>4		10 (9.0)
Was Impella planned pre-operatively?	111	33 (29.7)
Pre-op median TTE EF (%)	99	22.5 [19, 33]
Discharge median TTE EF (%)	95	32.5 [26, 43]
Duration of Impella support (days)	111	7.0 [5.0, 9.0]

Preoperative EF details were unavailable for 12 patients. EF data was unavailable for up to 16 patients.

Impella support was placed preoperatively in 4 patients, intraoperatively in 105 patients, and postoperatively in 2 patients. The preferred device insertion approach was direct aortic access (*n* = 106), while right axillary artery cannulation was used in 5 cases.

All 111 patients underwent CABG with Impella support during the same hospitalization. Impella 5.5 was used in 82 patients (73.9%), and Impella LD in 29 patients (26.1%).

Impella placement was planned preoperatively in 33 patients (29.7%). The median duration of Impella support was 7 days (IQR, 5–9).

### Bleeding outcomes

Of the 108 patients eligible for analysis, 2 (1.9%) met the study-specific bleeding criteria. Three patients were excluded because they did not survive beyond 24 h after their final surgical procedure.

### Postoperative outcomes

Postoperative outcomes are summarized in Table 4. Overall 30-day mortality was 10.8% (*n* = 12), and 1-year mortality was 25.2% (*n* = 28). Intraoperative survival was 100%. No statistically significant difference in 30-day or 1-year mortality were observed according to operative time points (2018–2020 vs. 2021–2025), performance of concomitant procedures, planned versus unplanned Impella use, or presence of advanced cardiogenic shock at admission.

**Table 4:** Summary of clinical outcomes by patient groups.

Characteristics	*N*	*N* (%) or [IQR]	*N* (%) or [IQR]	*p*-value
Operative Time Points		2018–2020 (*N* = 32)	2021–2025 (*N* = 79)	
30 Day Mortality	111	3 (9.4)	9 (11.4)	>0.9
1 Year Mortality	111	7 (21.9)	21 (26.6)	0.6
Concomitant Procedures		No (*N* = 92)	Yes (*N* = 19)	
30 Day Mortality	111	10 (10.9)	2 (10.5)	1.0
1 Year Mortality	111	22 (23.9)	6 (31.6)	0.56
Impella Placement Strategy		Planned (*N* = 33)	Unplanned (*N* = 78)	
Length of Hospital Stay (days)	111	26 [19, 33]	22 [15, 29]	0.12
30-day Mortality	111	4 (12.1)	8 (10.3)	0.7
1 Year Mortality	111	8 (24.2)	20 (25.6)	0.9
Advanced Cardiogenic Shock at Admission		Yes (*N* = 14)	No (*N* = 97)	
30-day Mortality	111	2 (14.3)	10 (10.3)	0.65
1-Year Mortality	111	3 (21.4)	25 (25.8)	1.0

### Hospitalizations

The median length of hospital stay was 23 days (IQR 16, 31). Rehospitalization data was available for 95 patients. At 6 months, 50 patients (52.6%) had freedom from rehospitalization, 28 patients (29.5%) had one, and the remainder experienced ≥ 2 rehospitalizations. By 1 year, 43 patients (45.3%) had no rehospitalizations, 29 patients (30.5%) had one, and the remaining experienced multiple rehospitalizations, with 7 (7.4%) requiring ≥ 5 readmissions.

### Renal and cardiac outcomes

During the index hospitalization, 27 patients required temporary renal replacement therapy. Among the 92 patients with available post-discharge renal follow-up, 80 (87%) did not require hemodialysis (HD), 8 (8.7%) required temporary HD for up to 3 months, and 4 (4.3%) progressed to permanent HD. Patients with preexisting CKD ≥ stage III were significantly more likely to require post-discharge HD than those without CKD ≥ stage III (46.7% [7/15] vs. 6.5% [5/77], *p* < 0.001).

Among patients with CKD ≥ stage III, 4 of 15 (26.7%) required temporary HD and 3 of 15 (20%) required permanent HD, compared with 4 of 77 (5.2%) and 1 of 77 (1.3%), respectively, among patients without CKD ≥ stage III.

LVEF improved during hospitalization. Median EF increased from 22.5% (IQR 18.8–32.5) preoperatively to 32.5% (IQR 26.3–42.5%) at discharge (*p* < 0.001).

### Postoperative complications

30-day postoperative complications included cerebral vascular accident (CVA) in 5 patients (4.5%) confirmed by neuroimaging and acute kidney injury (AKI) in 58 patients (52.3%). During follow-up, 1 patient (0.9%) ultimately required LVAD implantation, and 2 (1.8%) underwent heart transplantation.

### Survival analysis

Kaplan–Meier survival analysis demonstrated survival over the follow-up period, including 110 of 111 patients with available follow-up data (Figure 1). The discriminatory performance of the STS PROM score was evaluated using receiver operating characteristic analysis (Figure 2). The area under the curve (AUC) was 0.71 (95% CI, 0.61–0.8), indicating acceptable prediction for mortality. The mean STS PROM was 15.7%.

**Figure 1:**
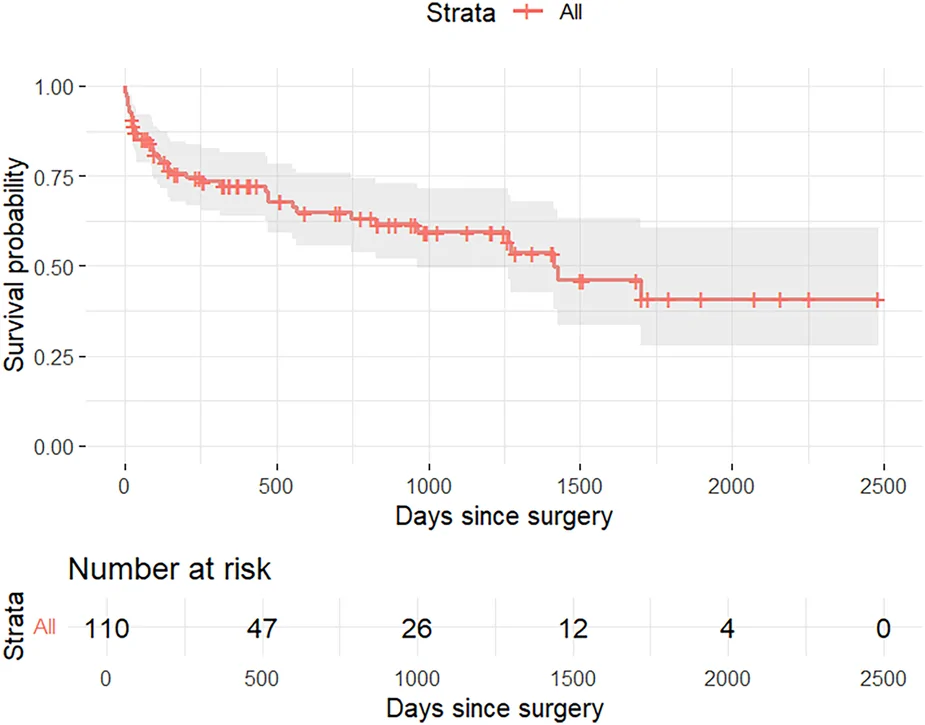
Kaplan–Meier survival curve demonstrating all-cause mortality. Survival analysis included 110 of 111 patients with available follow-up data.

**Figure 2:**
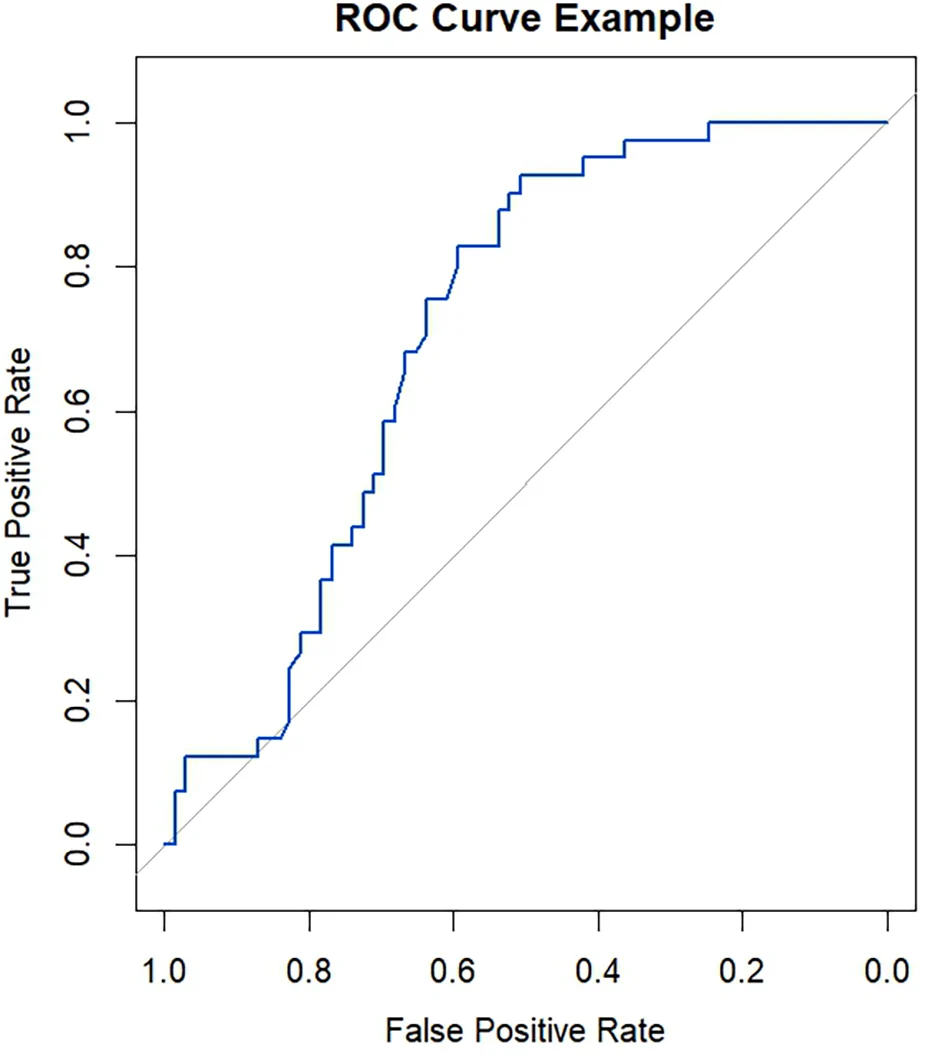
Discriminatory performance of the STS PROM score for the study cohort.

## Conclusion

In this single center, retrospective study of high-risk CABG patients with severely reduced LVEF (median EF 22.5% pre-operatively), Impella support was associated with a 30-day survival of 89.2% and 1-year survival of 74.8%. Despite a cohort characterized by profound ventricular dysfunction, substantial operative risk (mean STS PROM 15.7%), and a subset of patients presenting with advanced cardiogenic shock (12.6%), observed 30-day mortality was lower that historically reported outcomes among similarly high-risk surgical populations. Prior studies of patients with severe ischemic cardiomyopathy, cardiogenic shock, or requiring perioperative MCS support have reported operative or early mortality rates ranging from approximately 17% to >50%, depending on baseline clinical and hemodynamic status and perioperative ventricular dysfunction (10–12). The median hospital length of stay was 23 days, reflecting the complexity of this cohort, as evidenced by the STS PROM and inclusion of patients requiring preoperative stabilization for advanced cardiogenic shock, among other factors.

More recent registry data from two cohorts report 30-day mortality rates ranging from 8% to 19% among patients with EF ≤35% undergoing CABG (13, 14). Both cohorts included selective use of MCS with perioperative intra-aortic balloon pump (IABP) utilization reported in 30% and 26.3% of patients, respectively, though outcomes stratified by MCS use were not reported. Neither study reported STS risk-adjusted comparisons; the latter instead reported a EuroSCORE II of 7.48 ± 5.76, indicating a high-risk cohort with substantial variability in predicted operative mortality (13, 14). The wide variation in observed mortality across these cohorts highlights the diversity of this high-risk population and limits predictability of outcomes between studies. In this context, the lower observed mortality in our cohort suggests that Impella-supported CABG may achieve improved early survival even among patients with advanced ventricular dysfunction and a high-risk clinical profile.

This interpretation is further supported by risk stratification within our cohort. Despite a mean STS PROM of 15.7%, observed mortality was lower than expected based on standard surgical risk modeling. The discrepancy between predicted and observed mortality may reflect the potential benefit of perioperative ventricular unloading and hemodynamic stabilization in this high-risk population. Furthermore, patients presenting in advanced cardiogenic shock underwent expedited revascularization despite markedly higher lactate levels, greater support requirements, and shorter time to surgery, yet achieved comparable mortality outcomes to patients without advanced cardiogenic shock Similarly, planned and rescue Impella strategies demonstrated comparable outcomes; this aligns with prior studies reporting comparable survival between planned and rescue Impella in high-risk cardiac surgery (15, 16). Although these subgroup analyses are limited by small sample sizes, the observed outcomes support the feasibility of CABG with perioperative Impella support as a management strategy for patients with severe LV dysfunction and elevated operative risk.

Physiologically, Impella support unloads the LV, improves coronary perfusion pressure, and enhances systemic perfusion, mechanisms supportive of patients with severe cardiomyopathy undergoing CABG. Previous studies have demonstrated improved end-organ perfusion and myocardial recovery with Impella use in similar populations (15–18). Our results build on these observations by demonstrating favorable survival outcomes even in a cohort with a large proportion of unplanned device use, suggesting that Impella may extend surgical candidacy to patients with profound LV dysfunction who might otherwise be deemed inoperable.

Consistent with these physiologic benefits, Impella support in our cohort was associated with meaningful improvements in cardiac function. Median LVEF increased by approximately 10% from preoperative reports to discharge, suggesting early myocardial recovery during the index hospitalization. Although improvements in LVEF following revascularization alone is well recognized (19, 20), the magnitude and early timing of recovery observed in this cohort are notable but require further validation in larger, prospective cohorts. The retrospective nature of this study does not account for the extent of CAD, the quality of the target vessels, the chronicity of decreased EF prior to surgery, the degree of LV enlargement, and the extent of myocardial viability, which can all independently affect both mortality and myocardial recovery. Patient treatment was subject to multi-disciplinary evaluation via our heart team, which included CT surgery, heart failure, and interventional cardiology.

Several limitations warrant consideration. The retrospective design and absence of a matched control group limit conclusions regarding causality. In addition, subgroup analyses, including patients undergoing concomitant procedures and those receiving planned Impella support were limited by small sample sizes, reducing statistical power to detect clinically meaningful differences in outcomes. We underscore that this patient population does not reflect a general coronary revascularization population. Rather, the study represents a highly selected group of patients with severe LV dysfunction and elevated operative risk, identified through multidisciplinary heart team evaluation and concurrent assessment of surgical candidacy. Consequently, identification of an appropriate control group is inherently challenging as MCS was selected based on clinical judgement in patients considered to be at substantial risk for perioperative hemodynamic instability. A comparison with lower-risk patients not requiring support would introduce significant confounding, while withholding indicated MCS in similarly high-risk patients would raise ethical concerns. Additionally, inclusion of patients requiring postoperative Impella support introduces heterogeneity in the timing of device initiation. However, these patients were intentionally included as a part of a perioperative CABG cohort, as postoperative escalation to MCS reflects the spectrum of hemodynamic instability and complex clinical decision-making that guides Impella use before or during surgery. Additionally, long-term functional outcomes beyond 1 year were not consistently available. Despite these limitations, the consistency of our results with published outcomes supports the generalizability of our findings.

In conclusion, Impella-supported CABG in high-risk, profoundly reduced EF patients was associated with acceptable 30-day and 1-year survival rates superior to what would have been predicted by STS, favorable early recovery of ventricular function, and limited progression to permanent renal failure. Outcomes were unaffected by unplanned placement, suggesting flexibility in clinical applications. Collectively, these findings support the role of Impella as an effective adjunct in complex coronary surgeries and indicate that its use may expand surgical candidacy among patients with advanced LV dysfunction, including select patients presenting in advanced cardiogenic shock, who would often be considered beyond the scope of surgical revascularization with traditional pharmacologic and IABP rescue. Further studies are warranted to define optimal patient selection, timing of support, and long-term functional outcomes.

## Data Availability

The raw data supporting the conclusions of this article will be made available by the authors, without undue reservation.
